# Archaea, from obscurity to superhero microbes: 40 years of surprises and critical biological insights

**DOI:** 10.1042/ETLS20180022

**Published:** 2018-12-14

**Authors:** Nicholas P. Robinson

**Affiliations:** Department of Biomedical and Life Sciences, Faculty of Health and Medicine, Lancaster University, Lancaster LA1 4YG, U.K.

**Keywords:** archaea, Asgard, CRISPR, evolution

## Abstract

This issue of *Emerging Topics in the Life Sciences* highlights current areas of research in the field of archaeal biology and the following introductory editorial sets the stage by considering some of the key developments over the last four decades since the initial identification of the archaea as a unique form of life. Emerging topics from this vibrant and rapidly expanding field of research are considered and detailed further in the articles within this issue.

At the end of the 1960s, a (now-outdated) five-kingdom system was proposed to classify all forms of cellular life, dividing organisms into either the prokaryotic monera (the eubacteria, more commonly referred to as bacteria) or alternatively into the eukaryotic divisions of the fungi, protists, plants and animals [1,2]. The name eukaryote is derived from the Greek words ‘eu’ meaning ‘true’ and ‘karyon’ meaning ‘kernel’ or ‘nut’ in reference to the nucleus, the membrane-bound feature of these organisms where the genetic material is stored. As the name suggests, this compartment is absent in prokaryotic cells. Furthermore, these rudimentary organisms also lack the more complex cellular features associated with the eukaryotes, such as endomembrane systems including the Golgi apparatus, and membrane-bound organelles such as mitochondria (and the chloroplasts in plants) [3]. However, having effectively separated all biological life into these taxonomic divisions, the biological community could not have predicted that an entirely new category would be eventually be required to account for a unique form of life, the archaea.

In what is arguably one of the most unexpected biological discoveries of the 20th century, a publication at the end of the 1970s astounded the scientific community by proposing an entirely new division of life, initially referred to as the Archaebacteria and then subsequently renamed as the Archaea (derived from the Greek word ‘archaios’ meaning ‘archetypal’, ‘primitive’ or ‘ancient’) [4]. These organisms were originally distinguished from other microbes by phylogenetic approaches developed by Carl Woese, George Fox and colleagues who initially used the RNA sequences of components of the ribosome (the machinery that manufactures proteins from the genetic code in cells) to examine the evolutionary relationships between a wide sample of divergent bacterial species [5]. The breakthrough arose when Woese and colleagues examined the ribosomal RNA sequences from an unusual family of microbes known as the methanogens; anaerobic species that generate methane during the cellular processes used by the cells for the generation of energy. Woese recognised immediately that the ribosomal RNA sequences of these species were completely distinct from those of the other microbes and animatedly noted to his colleagues that these organisms were clearly not bacteria but instead represented an entirely new form of life. This conceptual departure was so radical that initially the work was met with great resistance and scepticism by some in the scientific community, but with time and further investigation it became clear that biological entities could not simply be divided broadly into two divisions of prokaryotes and eukaryotes, and a three ‘domain’ categorisation was proposed encompassing the eukaryotes, bacteria and the newly discovered archaea. Indeed, reconstructions of the resultant tripartite tree of life suggested that while the archaea were unique organisms, distinct from both the bacteria and eukaryotes, they were more closely related to the eukaryotes than the bacteria, sharing a common phylogenetic ancestry with the more complex life forms [6].

In the current age of social media and the associated rapid dissemination of news and scientific discovery [7], it seems likely if such a breakthrough had been revealed today there would be considerable global interest and collective awareness of these discoveries. However, although the archaea have become increasingly prominent in the public eye, it is common to meet individuals outside the immediate scientific communities who are uninformed or even completely unaware of these organisms. Nevertheless, in just four decades since their discovery, considerable research efforts focused on archaeal biology have been instrumental in advancing our understanding of a wide variety of critical biological processes, some of which have led to important mechanistic insights and biotechnological innovations [8]. Knowledge of the Archaea has also improved our understanding of ecosystems associated with a wide variety of important biological niches with potential far-reaching impact in terms of the environment and world economy [8]. Furthermore, studies of archaeal biology have provided insights into how life emerged and diversified on Earth and our understanding of the extremophilic archaea has even helped to redefine the parameters in the search for life beyond this planet [9,10].

In parallel with the increasing general awareness of the Archaea, descriptions of these organisms have more recently appeared in general scientific textbooks and are now also mentioned in blogs, social media sites and general press releases, and consequently, an appreciation of these organisms is beginning to enter the collective consciousness of the general population. Notably, public interest in this subject area has recently been piqued by the discovery of an entirely novel superphylum of archaea, the Asgard archaea [11,12]. Named after the Norse gods including Loki, Thor, Odin and Heimdall, these archaeal species have led to a departure in our understanding of how some cellular features characteristic of eukaryotic cells emerged, thereby provided us with tantalising hints regarding the evolution of more sophisticated forms of life [13]. The Lokiarchaeota were the first of the Asgard species to be identified in cold marine sediments not far from a deep-sea hydrothermal feature known as Loki's castle. It seemed fitting that these organisms were named after the shapeshifting Norse god of mischief as the metagenome assemblies from the study appeared to encode a variety of cellular features that were previously believed to be exclusively eukaryotic innovations. Soon, other related organisms were discovered in different ecological niches and each species was named after a Norse god, serendipitously coinciding with a glut of Hollywood blockbusters featuring superhero incarnations of these mythological deities. It has therefore become clear that the Asgard archaea represent a broad superphylum of organisms existing in varied ecological niches across the globe [12]. Currently, our understanding of Asgard species is entirely gleaned from bioinformatic interpretation of metagenomic assemblies, as these microbes are yet to be cultured. The Asgardian species genetically encode eukaryotic-like components of the endomembrane systems, small GTPases, membrane trafficking machineries, vesicle biogenesis proteins, post-translational protein modification systems and cytoskeletal components that are absent in non-Asgardian archaeal cells [12]. Indeed, the extensive phylogenetic analyses of these Asgard metagenomes seemingly suggest an intimate relationship with the eukaryotes, indicating that eukaryotic organisms likely arose from an Asgard-like ancestor, or an as-yet undiscovered sister group of these recently identified Archaea [13–15]. It should be noted, however, that the topology of the ‘tree of life’ remains an active and intensely contested area of research, hypotheses and discussion [16].

Over the last 40 years, archaeal organisms have been investigated by a growing cohort of dedicated research groups from around the world leading to advances in our broad understanding of the ecology and metabolism of these fascinating organisms. Investigations of archaeal model systems have also unveiled valuable details regarding the genetics, biochemistry and associated molecular mechanisms of a wide variety of fundamental cellular processes such as cell division [17], protein homeostasis [18], DNA replication and genomic repair [19]. Studies of the transcription and translation apparatus from archaea have also proved invaluable in our understanding of these fundamental processes central to all life [20,21]. Given the close phylogenetic relationship between the archaea and the eukaryotes, many of these studies have aided our understanding of how the equivalent mechanisms operate in our own cells. In many cases, these studies have taken advantage of the intrinsic biochemical robustness of the proteins and complexes associated with the thermophilic archaea [22]. These archaeal homologues frequently prove to be more experimentally tractable than the more complex and less stable eukaryotic counterparts, and this is reflected by the several thousand archaeal protein X-ray crystal structures that have been deposited to the Protein Data Bank (PDB). Indeed, structural studies of the archaeal homologous of a wide variety of macromolecular assemblies including the RNA polymerases [23], DNA replication machineries [19], ribosomes [24], proteasomes [25] and chaperonins [26] have all led to advancements in our understanding of the parallel processes in complex eukaryotic cells.

The ongoing study of archaeal biology will also no doubt lead to discoveries that will unlock future scientific knowledge and economic potential. We now take for granted the high-fidelity thermostable polymerases that are utilised in the polymerase chain reaction [27]. These enzymes remain key tools in molecular biology laboratories and represent an essential workhorse in the fields of environmental microbiology, metagenomic assembly medicine and forensic science. Thermophilic, denaturation-resistant biocatalysts including proteases, lipases, lignases, cellulases, xylanases, dehydrogenases and esterases are also employed in a range of industrial processes to significantly improve productivity or reduce cost [28,29]. It is clear that the archaea offer a largely unexplored reservoir of other biotechnologically useful enzymes, many of which have significant benefits. Indeed, it is worth emphasising that the recent development of the high-profile CRISPR-Cas technology has origins routed firmly in studies of previously undiscovered prokaryotic adaptive immune systems, utilised by both archaea and bacteria to recognise foreign DNA to provide protection from viruses and other assaults to the host genome [30]. This technology has revolutionised our ability to perform genome editing in almost any organism including, most controversially, humans. Understandably, these developments have captured the attention of the wider general public and awareness of the technology, and its implications for genetic modification have spread rapidly through modern media dissemination. CRISPR stands for Clustered Regularly Interspersed Short Palindromic Repeats and refers to unusual palindromic repeated elements that were first described by Mojica as short regularly spaced repeats in the halophilic archaea *Haloferax mediterranei* [31]. Similar repeats had also been observed by Ishino et al. [32] in the bacterium *Escherichia coli*, and Mojica and others eventually determined that these repeats represented a repository for viral and other foreign DNA elements in an adaptive immune system that protects the host genome from invading genetic material. Considerable and incremental research efforts by many bioinformaticians, microbiologists and structural biologists (reviewed in ref. [33]) have resulted in a clear understanding of how this system operates, driven by the Cascade (Cas) protein machinery, and how these systems could be appropriated as a genome editing tool [34–39]. In recognition of their seminal contributions to this field Doudna, Charpentier and Šikšnys were recently awarded the prestigious Kavli prize in nanoscience. This technology is also predicted to win a Nobel Prize in the near future, and the story of the discovery and development of this radical biomolecular tool provides a clear example of how far-reaching advances in medicine and biotechnology can arise from fundamental research in simple prokaryotic life forms.

It should be stressed that while the Archaea were for many years often perceived as particularly unusual and obscure organisms, existing exclusively in extreme ecological environments such as the deep-sea hydrothermal vents or hypersaline lakes [40], these organisms are considerably more diverse and adaptable and not restricted to hostile habitats. Indeed, it is now widely accepted that these microbes have occupied almost every imaginable ecological niche in the biosphere and contribute critically to biomass and global nutrient cycling in our oceans, soils and other ecologically important environments [41,42]. For example, archaea have recently been shown to play important contributions to ammonia oxidation, the first step of nitrification in the nitrogen cycle [43]. Furthermore, when considering the biological generation of methane, a critical greenhouse gas associated with global warming, it is worth emphasising that this is solely the metabolic product of the methanogenic archaea [43]; pertinently, it was the phylogenetic classification of these unique methanogenic organisms, originally discovered by Ralph Wolfe, that led his co-worker Woese to first recognise and define the archaeal division of life itself [4].

There is also increasing recognition of the importance of archaea in the microbiomes of both plants and animals [44]. It is becoming clear that the presence of important archaea in microbial communities has been significantly underrepresented to date owing to the insensitivity or lack of specificity of previous detection methods. Now, with advanced detection methodologies, new archaeal populations have been identified in a range of microbiomes including organisms in plant root systems and also as in the human biome on the skin, nasal cavity, lungs and digestive system [44]. While, to date, archaea have not been found to be the causative agent of any plant or animal disease, it is clear that close interactions and transactions occurring between archaeal and bacterial microbes within a community may have critical biological ramifications such as modulating or potentiating the virulence of an infection or altering the availability of nutrients in the rhizosphere of a plant [45]. It therefore seems probable that the study of archaeal population interactions within microbiomes will have repercussions in important areas such as food sustainability and maintaining a healthy and disease-free human microbiome. In addition, it is worth considering that archaeal species are also likely to harbour unexplored reservoirs of novel antibiotic molecules and systems, perhaps providing a solution to the alarming global increase in the resistance of pathogenic bacteria to the majority of the current generation of antibiotic agents [46–48].

As we now enter the fifth decade of biological investigation of archaeal organisms these widespread, but often overlooked, microbes have provided new avenues of biological study, improving our understanding of ecosystems, microbiomes and fundamental cellular mechanisms. With the increasing awareness of the existence and biological importance of the Archaea, the number of researchers studying archaeal biology is steadily increasing, and ongoing and future study of this broad division of life offers exciting potential for novel discoveries.

This issue of *Emerging Topics in the Life Sciences* on the Archaea provides a snapshot of some of ongoing developments in archaeal biology, but many important studies and topics are not considered here due to space limitations. The spotlight on archaeal biology has never been brighter and this field offers great opportunities for the next generation of researchers.

## Abbreviations

Cas: cascade
CRISPR: Clustered Regularly Interspersed Short Palindromic Repeats
PDB: Protein Data Bank
